# Synaptic Characteristics from Homogeneous Resistive Switching in Pt/Al_2_O_3_/TiN Stack

**DOI:** 10.3390/nano10102055

**Published:** 2020-10-18

**Authors:** Hojeong Ryu, Sungjun Kim

**Affiliations:** Division of Electronics and Electrical Engineering, Dongguk University, Seoul 04620, Korea; hojeong.ryu95@gmail.com

**Keywords:** memristor, synapse device, neuromorphic computing, homogeneous resistive switching

## Abstract

In this work, we propose three types of resistive switching behaviors by controlling operation conditions. We confirmed well-known filamentary switching in Al_2_O_3_-based resistive switching memory using the conventional device working operation with a forming process. Here, filamentary switching can be classified into two types depending on the compliance current. On top of that, the homogeneous switching is obtained by using a negative differential resistance effect before the forming or setting process in a negative bias. The variations of the low-resistance and high-resistance states in the homogeneous switching are comparable to the filamentary switching cases. However, the drift characteristics of the low-resistance and high-resistance states in the homogeneous switching are unstable with time. Therefore, the short-term plasticity effects, such as the current decay in repeated pulses and paired pulses facilitation, are demonstrated when using the resistance drift characteristics. Finally, the conductance can be increased and decreased by 50 consecutive potentiation pulses and 50 consecutive depression pulses, respectively. The linear conductance update in homogeneous switching is achieved compared to the filamentary switching, which ensures the high pattern-recognition accuracy.

## 1. Introduction

Data usage required by new technologies, such as self-driving cars, Internet of Things (IoT), and Artificial intelligence (AI), has been increasing rapidly [1]. A low-power, highly integrated, and power-efficient computing system design is highly important for coping with big data. The existing von Neumann architecture with serial data processing has problems of bottlenecks between the central processing unit (CPU) and the memory. Additionally, the von Neumann structure is efficient for simple calculations [1], but is not suitable for complex data processing, such as image and voice analysis. Therefore, a new computing system is needed to process data efficiently. One solution was found in the human brain, in which more than 100 billion neurons communicate with other neurons through 100 trillion synapses, processing and storing information in an instant. The neurons and synapses are connected in parallel and perform memory operations, reasoning, and learning at the same time, even with a low power of about 20 W. The neuromorphic chip is the next-generation computing technology that mimics the way the brain works. There are several electronic devices that include transistors as neurons and memory as synapses [2,3,4]. Since artificial neurons and synapses are configured in parallel like the human brain, they can process more data with much less power than previous processors could. Neuromorphic technology is efficient, because data processing can be integrated at once, and energy consumption is reduced [2,3,4]. The neuromorphic computing is more suitable for complex tasks such pattern recognition [5]. The conductance in the synaptic device is used as synapses in neural networks, and back-propagation is commonly used for learning algorithms in hardware-based neuromorphic systems [6]. 

When an operation is performed in a neuromorphic chip, the conductivity weight in each synapse that corresponds to the strength of the connection between neurons can be updated by applied pulse stimulation. It is important to implement multilevel conductance by analog switching to provide synaptic functions in a neuromorphic chip. Resistive switching random access memory (RRAM) can act as a synapse in a neuromorphic chip because of its low-power operation [7], fast switching time [8], high-density integration [9], and multi-level cells (MLC) with analogue switching [10,11,12,13,14]. The various resistive switching characteristics are achieved using a dielectric material and metal electrodes [15,16,17,18,19,20,21,22,23,24,25,26,27]. Additionally, the switching type can be changed depending on the operation conditions, such as the current and voltage levels [28]. Typically, two types of resistive switching that are well known are filamentary switching and homogeneous switching [28]. The filament switching is an instantaneous conducting-filament formation in a critical electric field during the set process, which is accompanied by a sudden current jump. Therefore, it is difficult to obtain multi-level conductance values during the set process. On the other hand, homogeneous switching is usually performed by charge trapping and detrapping, in which gradual switching is a highly desirable property in the synapse device in neuromorphic engineering [29]. High-k dielectrics, such as HfO_2_ and Al_2_O_3_ deposited by atomic layer deposition (ALD), have the advantage of accurate controllability of the thickness with good uniformity for RRAM applications [30,31,32,33,34]. Several studies of Al_2_O_3_ dielectric film have been performed for resistive switching and synaptic properties [33,34,35]. However, studies on the homogeneous switching properties for neuromorphic computers in Al_2_O_3_-based RRAM devices have been few. 

In this paper, we obtained both filamentary and homogeneous switching by controlling device operation conditions. We emulate the bio-inspired synaptic characteristics, such as short-term plasticity (STP), including paired pulsed facilitation (PPF), potentiation and depression, and resistive switching characteristics in a Pt/Al_2_O_3_/TiN device. We closely investigated the homogeneous resistive switching in the trapping/detrapping region without the electroforming as well as the conventional filamentary switching operation in Al_2_O_3_ in terms of the variation and retention of high-resistance state (HRS) and low-resistance state (LRS), multi-level properties, and synapse emulation properties.

## 2. Materials and Methods

We fabricated Pt/Al_2_O_3_/TiN as a memristor device as follows. First, a 100-nm-thick TiN was deposited by DC sputtering on a SiO_2_/Si substrate as the bottom electrode. Next, an Al_2_O_3_ thin film was deposited by ALD on the TiN bottom electrode. We used Al(CH_3_)_3_ (TMA) and H_2_O as precursors for the Al_2_O_3_. The source temperature was set to 9 °C and the chamber temperature to 200 °C. One cycle for deposition of the Al_2_O_3_ layer consisted of TMA 0.2 s, purge gas 15 s, H_2_O 0.2 s, and purge gas 15 s injection. We repeated a total of 60 cycles for the target thickness of 7 nm. Finally, 100 nm Pt as the top electrode was deposited by E-beam evaporation. We measured the electrical properties in the DC sweep and transient modes using a semiconductor parameter analyzer (Keithley 4200-SCS and 4225-PMU ultrafast module, Solon, OH, USA). During the measurements, we applied the bias voltage and pulse to the Pt top electrode, and the TiN bottom electrode was grounded. The step voltage is 0.02 V for DC sweep mode. We performed X-ray photoelectron spectroscopy (XPS) depth analysis with a Nexsa (Thermo Fisher Scientific, Waltham, MA, USA) with a Microfocus monochromatic X-ray source (Al-Kα (1486.6 eV)), a sputter source (Ar^+^), an ion energy of 2 kV, a sputter rate of 0.5 nm/s for SiO_2_, and a beam size of 100 µm.

## 3. Results and Discussion

**Figure 1:**
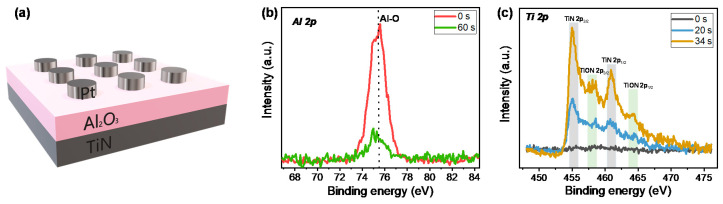
(**a**) Schematic drawing of the Pt/Al_2_O_3_/TiN device; (**b**) XPS Al 2p spectra; (**c**) Ti 2p spectra.

Figure 1a shows the schematic of the Pt/Al_2_O_3_/TiN structure. The circular Pt top electrodes with 100 μm diameter are on the Al_2_O_3_ insulator. To confirm the deposition of the ALD Al_2_O_3_ layer and interfacial layer between Al_2_O_3_ and the TiN bottom electrode, we investigated the XPS depth profile from the Al_2_O_3_ dielectric to the TiN bottom. Figure 1b,c show the XPS spectra of Al 2p and Ti 2p, respectively. The main peak is centered at about 75.5 eV, which corresponds to the Al–O bond at 0 s (Figure 1b). The Al peak intensity is relatively very low at the deep etching time (60 s), where the TiN layer is mainly exposed [36]. We closely checked the Ti 2p spectra to detect the TiON interfacial layer in Figure 1c. The Ti was not detected at 0 s, because of the Al_2_O_3_ layer on the surface. The peaks for TiN 2p_3/2_ and TiN 2p_1/2_ are centered at about 455 and 461 eV, respectively, at 20 and 34 s [37]. It is noted that we observed two peaks at around 458 and 464 eV for TiON 2p_3/2_ and TiON 2p_1/2_ at 34 s [37]. This suggests that the already-deposited Al_2_O_3_ layer could have been intermixed with the TiN layer during ALD Al_2_O_3_ dielectric deposition at 200 °C. 

**Figure 2:**
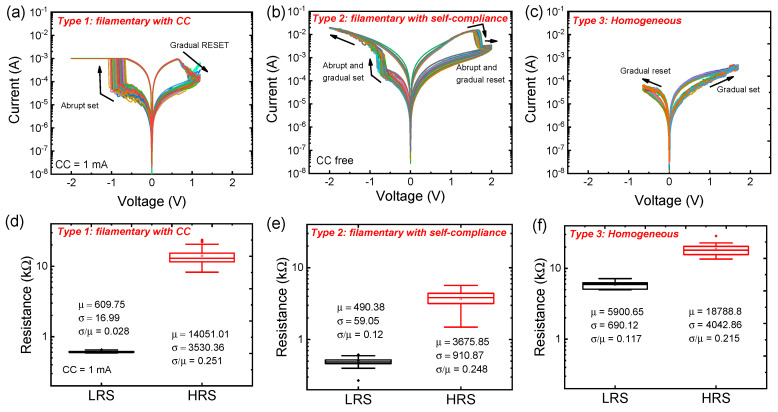
I–V characteristics of (**a**) filamentary switching with CC (type 1), (**b**) filamentary switching with self-compliance (type 2), and (**c**) homogeneous switching (type 3) in Pt/Al_2_O_3_/TiN device; high-resistance state (HRS) and low-resistance state (LRS) statistical distribution of (**d**) type 1, (**e**) type 2, and (**f**) type 3.

Figure 2a–c show three different I–V curves in the Pt/Al_2_O_3_/TiN device after the electroforming that is the activation process from the initial state in the Al_2_O_3_ dielectric (Figure S1). For the first switching mode (filamentary switching with compliance, type 1), we observed the typical bipolar resistance in which the set and reset processes occur in a negative bias and a positive bias, respectively. The set process in a negative bias is more favorable for good resistive switching than is the set process in a positive bias to use the TiON layer acting as an oxygen reservoir [38]. The TiON layer becomes thicker by accumulating oxygen during the positive bias, and then the oxygen vacancies are increased in the Al_2_O_3_ layer for the set process. It is noted that the conducting filament is formed by a so-called soft breakdown when the critical electric field is reached at the dielectric. Conversely, the oxygen in the TiON layer could return to the Al_2_O_3_ dielectric to reduce oxygen vacancies in the Al_2_O_3_ layer when a negative bias was applied to the top electrode for the reset process. In type 1, we used a compliance current (CC) of 1 mA for the forming and set processes (Figure 2a). The LRS and HRS can be adjusted by CC and reset stop voltage like many previous reports [39,40].

In type 2, the self-compliance is used for the set process without CC. When the current level reaches about 1 mA by the abrupt set process, the current gradually increases, indicating that the filament is still gradually created in the self-compliance region (Figure 2b). The self-compliance may occur by the TiON buffer layer or the oxygen reservoir layer acting as series resistance. The similar studies presenting self-compliance in filamentary switching type were reported [41,42,43,44,45]. The self-compliance mode that is close to the actual device operation (pulse switching) has the advantage of reducing the circuits related to current limiting. However, the variation (relative standard deviation, σ/μ) of LRS is slightly larger in the self-compliance current mode compared to the switching condition at a CC of 1 mA (Figure 2d,e). We will discuss more about the self-compliance effect on the synaptic behavior in the pulse switching mode. The homogeneous switching (type 3) can be obtained by using a negative differential resistance effect before the electroforming or set processes (Figure S1) [34,46]. The initial reset process in a negative bias occurs probably because of the abundant initial traps in the Al_2_O_3_ layer. This behavior could be associated with a sort of asymmetric complementary resistive switching [47,48]. Subsequently, a set process occurs in a positive bias. When a positive bias is applied to the top electrode, additional oxygen vacancies can be created in the Al_2_O_3_ dielectric. In another way, the change in conductivity can be explained by charge trapping/detrapping [28]. Additionally, the oxygen exchange at the interface between the Pt and oxide could make the homogenous switching behavior [47].

It is noted that the conductance changes gradually in homogeneous switching without CC in the Pt/Al_2_O_3_/TiN device. Furthermore, type 1 and type 2 can be changed to type 3 after the reset process. The transition from the filamentary type to the homogeneous type is shown in Figure S2. The DC sweep in a positive bias can increase the conductance. There are still significant oxygen vacancies left, even though the conducting filament is ruptured. Similarly, the coexistence of filamentary and homogeneous switching was reported in a different material system [28,49].

The σ/μ values of the LRS are 0.028, 0.12, and 0.017 for type 1, type 2, and type 3, respectively (Figure 2d–f). The compliance control in filamentary switching provides the best LRS variation. On the other hand, the σ/μ values of the HRS are 0.251, 0.248, and 0.215 for type 1, type 2, and type 3, respectively (Figure 2d–f). The variation of HRS values in three switching modes are not very different.

**Figure 3:**
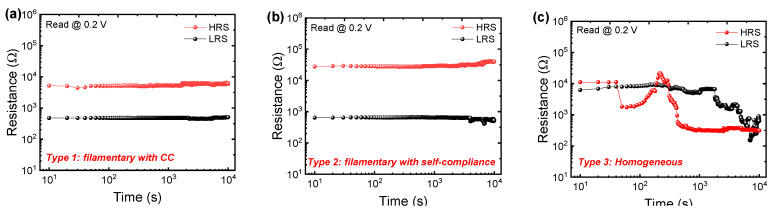
HRS and LRS resistance drift characteristics of (**a**) type 1, (**b**) type 2, and (**c**) type 3 in Pt/Al_2_O_3_/TiN device.

Next, we investigated the resistance drift characteristics for the three types in the Pt/Al_2_O_3_/TiN device. Figure 3a–c show the resistance change in the HRS and LRS for 10,000 s for type 1, type 2, and type 3, respectively. The change rates of LRS and HRS in filamentary switching with CC (type 1) for 10,000 s are 5.88% and 16.3%, respectively. The change rates of LRS and HRS in filamentary switching with self-compliance (type 2) for 10,000 s are 18.4% and 43.04%, respectively. The results indicate that the gradual conductance region by the self-compliance current in the LRS shows little unstable filament formation. The middle-resistance state (MRS) shows distinctively an unstable retention property, probably because the remaining oxygen vacancies are easily moved by partial reset (Figure S3). Additionally, the retention is not good for either LRS or HRS of homogeneous switching (type 3), whose switching mechanism is based on the charge trapping and detrapping (Figure 3c). Similarly, poor retention has been reported in homogeneous switching in RRAM devices composed of different materials. Therefore, type 3 is more suitable for the applications by the short-term memory effect, such as reservoir computing [50]. 

**Figure 4:**
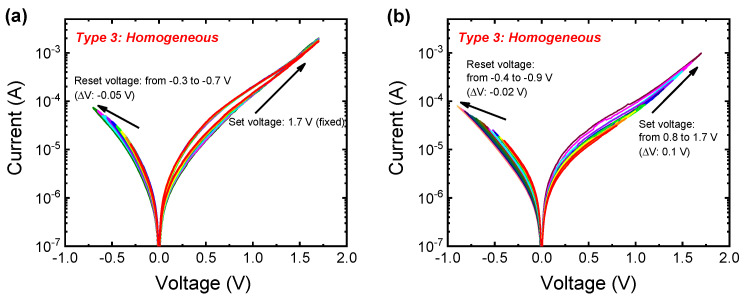
Multi-level states in the I–V curves by varying set voltage and reset voltage under the DC sweep in homogeneous switching of Pt/Al_2_O_3_/TiN device. (**a**) Reset stop voltage is controlled from −0.3 to −0.7 V at the fixed set voltage of 1.7 V; (**b**) set voltage from 0.8 to 1.7 V and reset voltage from −0.4 to −0.9 V.

The gradual switching could be an advantage when used as a synaptic device application despite the poor retention in the homogeneous switching in the Pt/Al_2_O_3_/TiN device. The multi-level states are demonstrated by controlling the set and reset stop voltages in Figure 4. Multiple reset processes occurred by varying the reset stop voltage from −0.3 to −0.7 V, with an incremental voltage of −0.05 V for each step when fixing the set voltage of 1.7 V (Figure 4a). Moreover, multiple set processes were controllable by changing the set stop voltage from 0.8 to 1.7 V with the incremental voltage of 0.1 V; subsequently, multiple reset processes were achieved by controlling the reset stop voltage from −0.4 to −0.9 V, with an incremental voltage of −0.02 V (Figure 4b).

**Figure 5:**
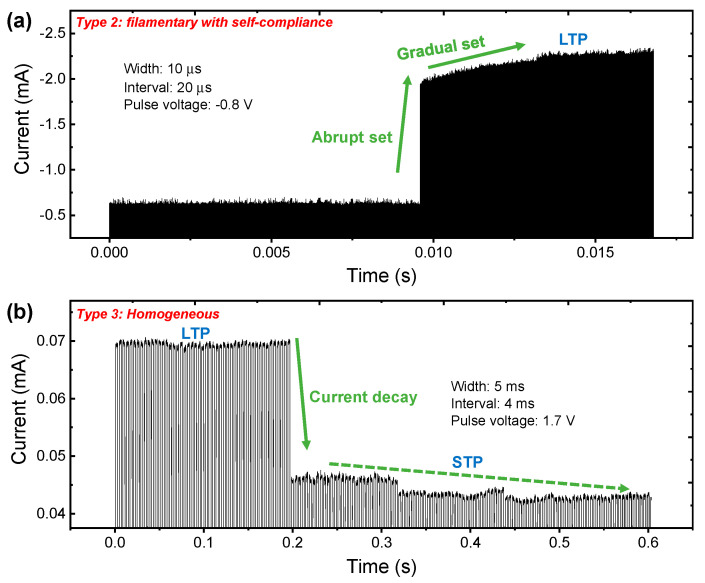
Current transient characteristics by repetitive pulse voltage inputs for (**a**) type 2 and (**b**) type 3 in Pt/Al_2_O_3_/TiN device.

To evaluate the synaptic performances, we investigated the short-term effects by the current response in pulse input. We confirmed the switching type after the DC sweep mode and applied repeated pulse input to the device. The compliance current function was not provided in the measurement system, so we characterized type 2 and type 3 in pulse transient characteristics. The long-term plasticity was observed in type 2 when the repeated pulse inputs (amplitude: −0.8 V; width: 10 μs; interval time between pulse inputs: 20 μs) were used in Figure 5a. When repeated stimuli accumulate in the device, breakdown occurs and the current increases rapidly in about 0.0096 s. This phenomenon is a kind of time-dependent dielectric breakdown (TDDB) in the oxide. The current is gradually increased after an abrupt increase that is well matched with the DC sweep mode in type 2. Conversely, in type 3, the short-term plasticity is observed when applying repeated pulses (amplitude: 1.7 V; width: 5 ms; interval time between pulse inputs: 4 ms) in Figure 5b. Here, we designed the long pulse interval for a short-term effect in type 3 compared to the long-term effect in type 2. The first pulse made the device from HRS to LRS. The LRS could not maintain, even though the consecutive pulse input was applied to the device. The current started to decay from 0.197 s. The STP can be explained as spontaneous decomposition when the oxygen vacancy-based conducting filament is weakly formed.

**Figure 6:**
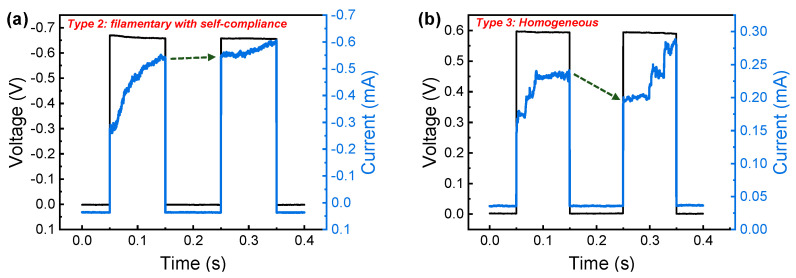
Paired pulsed facilitation (PPF) characteristics for (**a**) type 2 and (**b**) type 3 in Pt/Al_2_O_3_/TiN device.

Next, we used paired pulse facilitation (PPF) to confirm the long-term and short-term effects in two different switching modes (filamentary and homogeneous). PPF is a neural facilitation phenomenon in biological science. When two impulses were applied to a neuron, the post-synaptic potentials increased over the pre-synaptic potential, and the amount of increase was determined by the interval time between the two impulses. The PPF phenomenon in type 2 and type 3 was emulated, in which the width and interval of two pulse inputs were both 0.1 s. The current was not decayed in the filamentary switching, with good retention and a long-term memory effect when the pulse voltage of −0.65 V was applied to the device (Figure 6a). On the other hand, the current decay was distinctive when two inputs with the pulse voltage of 0.6 V were applied to the device (Figure 6b). However, when comparing the initial, intermediate, and last time of each pulse, we detected a larger current in the second pulse. Furthermore, it is confirmed that the PPF was exponentially decayed with interval time for type 3 (Figure S5). It is worthwhile to note that the synaptic dynamics such as those of a CBRAM type or diffusive memristor devices using Ag and Cu can be realized in our Pt/Al_2_O_3_/TiN device [51].

**Figure 7:**
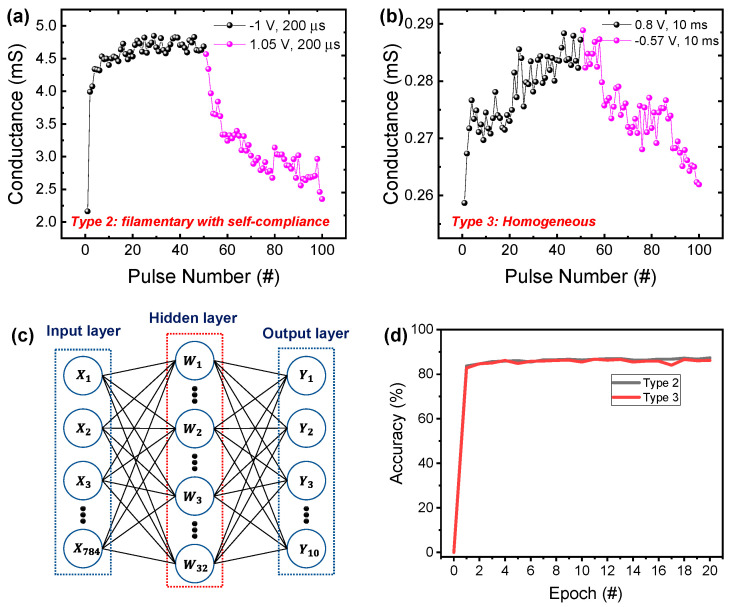
Potentiation and depression characteristics of (**a**) type 2 and (**b**) type 3; (**c**) schematic of neural network for pattern recognition accuracy; (**d**) pattern recognition accuracy for Fashion MNIST model when using the conductance of type 2 and type 3 as weight in neural network.

Next, we investigated the tendency of conductance (synaptic weight) update for the Pt/Al_2_O_3_/TiN device in filamentary and homogeneous switching. Figure 7a,b show the potentiation and depression curves for type 2 and type 3, respectively. For filamentary switching, we used the voltage of −1 and 1.05 V with the pulse width of 200 μs in the potentiation and depression, respectively (Figure 7a). We observed a gradual change after a sudden change in conductance by the first pulse, which is similar to the behavior observed in the DC sweep. Therefore, the gradual conductance change results from the self-compliance. For depression, from the beginning, we observed more gradual changes. Similarly, for homogeneous switching, the potentiation and depression were measured by applying the 0.8 and −0.57 V with pulses of 10 ms, respectively (Figure 7b). The nonlinearity and the conductance updated error (variation) of potentiation and depression were defined and calculated in Figure S3. To use and evaluate the controllable conductance values of the Pt/Al_2_O_3_/TiN device, we simply constituted the soft-based neural network simulation for Fashion MNIST pattern recognition. A neural network is composed of 3 layers (784 input neurons × 32 hidden neurons × 10 output neurons) and weight values of the synapse between each layer that can be adjusted, as shown in Figure 7c. We used the conductance value of the Pt/Al_2_O_3_/TiN device as the weight value of the synapse. Fashion MNIST has 10 different categories for pattern recognition; each image has 28 × 28 grayscale pixels, and its dataset has a training set of 60,000 examples and a test set of 10,000 examples. For simulation input values, 28 × 28 grayscale pixels are normalized and have values from 0 to 1. The pattern-recognition accuracy after 20 epochs using the conductance update of type 2 and type 3 were 87.34% and 86.17%, respectively (Figure 7d). The comparable accuracy of homogeneous switching results from the linear update, even though the variation (updated error) is worse than that of the filamentary switching with self-compliance (Figure S4). However, the poor retention properties in type 3 can have a negative impact on pattern recognition accuracy in the case of on-chip learning [6].

## 4. Conclusions

In summary, we demonstrated the coexistence of filamentary and homogeneous resistive switching in the Pt/Al_2_O_3_/TiN device by simply controlling the switching operation condition. We verified the ALD Al_2_O_3_ resistive switching layer and TiON interfacial layer by XPS analysis. The homogeneous switching showed HRS and LRS variation comparable to that of the filamentary switching. Moreover, the multi-level states in homogenous switching are achieved by the DC sweep mode and pulse mode, which is used for synaptic devices in neuromorphic applications. We confirmed comparable pattern-recognition accuracy in homogeneous switching by neural network simulation (784 × 32 × 10 layers) compared to that of the filamentary switching. The short time retention in LRS and HRS of homogeneous switching is used for short-term memory, which we verified by controlling pulse interval time. The current decay was also distinctively observed in PPF for homogeneous switching. 

## Supplementary Materials

The following are available online at https://www.mdpi.com/2079-4991/10/10/2055/s1, Figure S1: Negative differential resistance behavior in forming curve of Pt/Al_2_O_3_/TiN device; Figure S2: Transition from filamentary switching to homogeneous switching in Pt/Al_2_O_3_/TiN device; Figure S3: Drift characteristics of filamentary switching with self-compliance (type 2) in middle resistance states of Pt/Al_2_O_3_/TiN device; Figure S4: Nonlinearity of potentiation and depression curves. Figure S5: PPF value as a function of interval time.
