# Supplementary material for: Synaptic Characteristics from Homogeneous Resistive Switching in Pt/Al2O3/TiN Stack
Source: Nanomaterials (Basel). 2020 Oct 18;10(10):2055. doi: 10.3390/nano10102055 (PMC7603159; doi:10.3390/nano10102055)
Supplement: Supplementary file 1 [file nanomaterials-10-02055-s001.pdf]

nanomaterials

Supplementary Materials

Synaptic Characteristics from Homogeneous Resistive Switching in $Pt/Al_2O_3/TiN$ Stack

Hojeong Ryu and Sungjun Kim*

Division of Electronics and Electrical Engineering, Dongguk University, Seoul 04620, South Korea; hojeong.ryu95@gmail.com
*Correspondence: sungjun@dongguk.edu

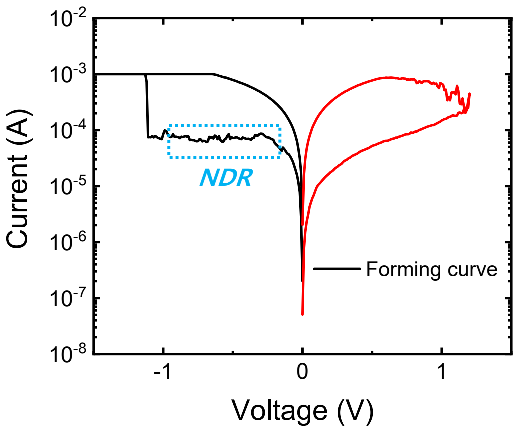

Figure S1. Negative differential resistance behavior in forming curve of $Pt/Al_2O_3/TiN$ device.

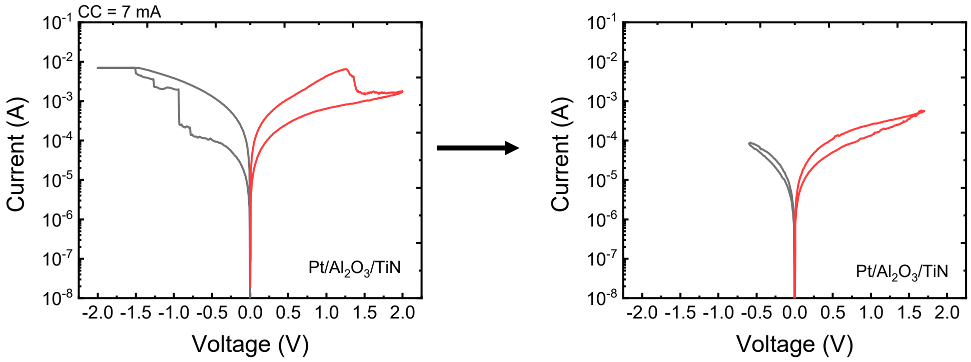

Figure S2. Transition from filamentary switching to homogeneous switching in $Pt/Al_2O_3/TiN$ device.

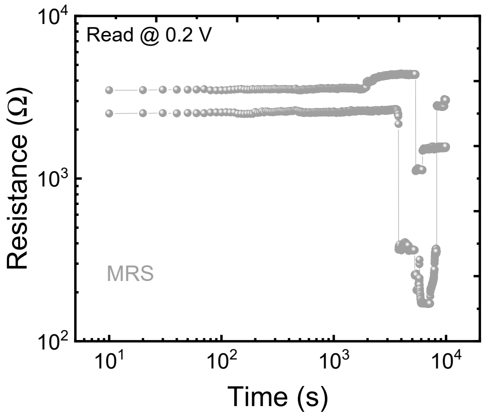

Figure S3. Drift characteristics of filamentary switching with self-compliance (type 2) in middle resistance states of Pt/Al_2O_3/TiN device.

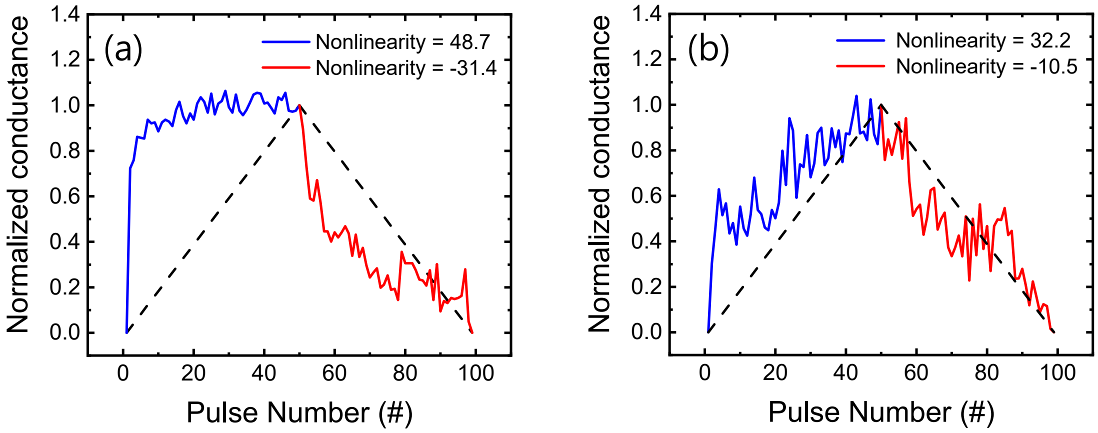

Figure S4. Nonlinearity of the potentiation and depression curves for (a) type 2 and (b) type 3. Nonlinearity is calculated by following equation.

$$\mathrm{NL} = \mathrm{average}\left(\left|\frac{G - G_{\mathrm{Linear}}}{G}\right| \times 100\right)$$

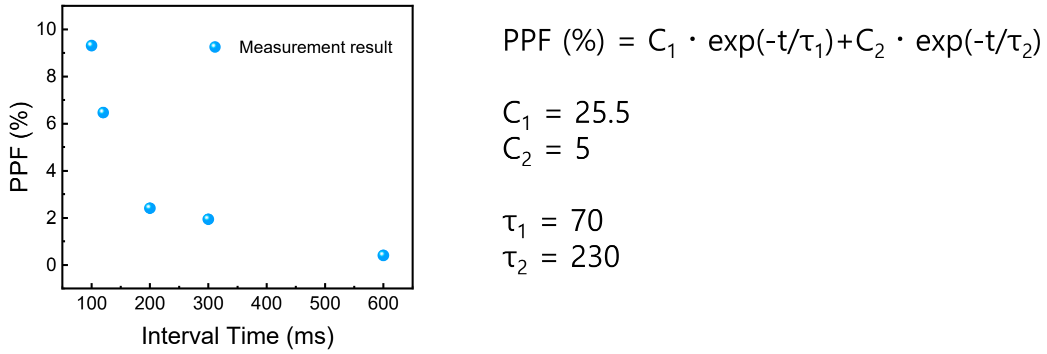

Figure S5. PPF index as a function of interval time.
